# Shrimp White Spot Viral Infections Are Attenuated by Organic Acids by Regulating the Expression of HO-1 Oxygenase and β-1,3-Glucan-Binding Protein

**DOI:** 10.3390/antiox14010089

**Published:** 2025-01-14

**Authors:** Ioan Pet, Igori Balta, Nicolae Corcionivoschi, Tiberiu Iancu, Ducu Stef, Lavinia Stef, Iuliana Cretescu

**Affiliations:** 1 Faculty of Bioengineering of Animal Resources, University of Life Sciences King Mihai I from Timisoara, 300645 Timisoara, Romania; ioanpet@usvt.ro (I.P.);; 2 Bacteriology Branch, Veterinary Sciences Division, Agri-Food and Biosciences Institute, Belfast BT4 3SD, Northern Ireland, UK; 3 Academy of Romanian Scientists, Ilfov Street, No. 3, 050044 Bucharest, Romania; 4 Faculty of Management and Rural Development, University of Life Sciences King Mihai I from Timisoara, 300645 Timisoara, Romania; tiberiuiancu@usvt.ro; 5 Faculty of Food Engineering, University of Life Sciences King Mihai I from Timisoara, 300645 Timisoara, Romania; ducustef@usab-tm.ro; 6 Department of Functional Sciences, Faculty of Medicine, Victor Babes University of Medicine and Pharmacy, 2 Eftimie Murgu Square, 300041 Timisoara, Romania; iuliana.cretescu@umft.ro

**Keywords:** white spot syndrome, shrimp, natural antimicrobials, organic acids, AuraAqua

## Abstract

The absence of efficient on-farm interventions against white spot syndrome viral (WSSV) infections can cause significant economic losses to shrimp farmers. With this exploratory study we aimed to test, both in vitro and in vivo, the efficacy of an organic acid mixture (Aq) against WSSV infections in shrimp. In vitro, using shrimp gut primary cells (SGP), 2% Aq significantly reduced WSSV infection and the amounts of H_2_O_2_ released but had no impact on CAT and SOD expression. In vivo, in a shrimp challenge test, 2% Aq significantly downregulated the expression of proteins involved in WSSV virulence, such as the lipopolysaccharide-β-1,3-glucan-binding protein (LGBP) and the TLR signalling pathway (LvECSIT), and increased the expression of HO-1 oxygenase. Additionally, at 2% Aq, the expression of the digestive-related enzyme carboxypeptidase B was upregulated in the gut, alongside a significant decrease in IL-22 expression, a cytokine usually increased during WSSV infection in shrimp. A low mortality rate (7.33%) was recorded in infected shrimp treated with 2% Aq compared to the 96.66% mortality in the absence of Aq. The peritrophic membrane (PM) was proven essential to ensure Aq efficacy, as the infected and treated PM deficient shrimp (PM−) had a mortality rate of 27.8%, compared to only 9.34% mortality in the infected shrimp at 2% Aq and in the presence of PM (PM+). Aq significantly increased the expression of mucin-1, mucin-2, mucin-5AC, mucin-5B, and mucin-19 in both PM+ and PM− shrimp. Conclusively, organic acid in mixtures can protect farmed shrimp against WSSV infection and increase their survivability through a mediated gut health effect which includes resistance to oxidative stress and improved immunity.

## 1. Introduction

Aquaculture has become one of the world’s most important and most rapidly developing sectors of the global food industry, particularly in the Asia–Pacific region due to the demand in high quality protein [1]. The Pacific white shrimp (*Litopenaeus vannamei*) represents over 70% of the world’s crustacean production, is worth above USD 18 billion, and employs over a million people [1,2,3]. For instance, in 2021, over USD 33 billion was estimated in global shrimp exports, contributing greatly to global food security and economic development [4]. The economic implications of white spot syndrome virus (WSSV) are profound, shaping the research priorities in shrimp aquaculture, including vaccination, and the integration of herbal and probiotic immune enhancers to overcome these high-cost barriers. Moreover, because the global shrimp production now exceeds 5 million tons per year [5], as reported by the Food and Agriculture Organization of the United Nations (FAO), it is noteworthy to provide robust, economically viable solutions to mitigate and combat WSSV and to maintain the viability of this important industry [1].

WSSV infects a diverse range of crustacean hosts, including the commercially important shrimp species *L. vannamei* and *Penaeus monodon* (≈90% of the market), but it can also infect crabs as well crayfishes [6,7,8,9]. The virus targets tissues of both ectodermal and mesodermal origin, with a specific affinity for the hepatopancreas of shrimp, where the host exhibits clinical symptoms such as lethargy, a discoloured exoskeleton, and pronounced white spots on the carapace [7,9,10]. Of note, the hepatopancreas, a vital organ for shrimp immune function, serves as the primary battleground in the resolution against WSSV, mediating both metabolic and immune responses [7].

Several biologic compounds providing high efficacy against WSSV have been recently revealed, including baicalein from *Scutellaria baicalensis* (SBEE) and coumarin derivatives (P13) [5,11]. Baicalein, at concentrations of 100 μM, has demonstrated a 66.7% effectiveness in reducing WSSV genome copy numbers in infected shrimp larvae, while also upregulating immune genes like Toll-4, Dorsal, and AMPs. This dual mechanism restricts viral replication, statisticallty reduces viral loads in vivo, and remarkably blocks the horizontal transmission of WSSV between hosts by enhancing host immunity, making it a viable candidate for inclusion in feed additives [11]. Similarly, P13 [N-(4-methyl-2-oxo-2H-chromen-7-yl)acetamide], a synthesized coumarin derivative, at an effective concentration of EC_50_ < 12 mg/L, has shown an antiviral response of over 90% in *L. vannamei* [5]. Following larvae infection with WSSV, treatment with P13 resulted in a notable viral inhibition and lower mortality rate of 45% compared with the control DMSO, which had a mortality rate of 80% at 120 hpi [5]. Plant-derived compounds like moringa (*Moringa oleifera*) leaf extract and olive (*Olea europaea*) leaf extract (OLE) have also gained importance for their immunomodulatory effects [12,13]. At the hepatopancreatic level, moringa leaf extracts at 0.5–1, including leaf powder at 25 g/kg, significantly enhanced the expression of immune genes such as alpha-2-macroglobulin (α2M), integrinß, LGBP, and peroxinectin (PX) [12]. Again, high doses of OLE improved both biochemical (total protein and glucose) and immunological parameters (OxH, SOD, PO activity, and clotting time), effectively supporting shrimp survival by up to 65% under viral challenges compared to the control [13]. Another promising compound, cuminaldehyde, achieved an 89% inhibition rate of WSSV replication at 40 μmol/L after 72 hpi and demonstrated remarkable potential in limiting horizontal transmission [14].

AuraAqua (Aq), a mixture of organic acids and other biological compounds, has been previously identified as efficient in preventing parasitic and bacterial infections in crustaceans [15]. Unlike conventional antibiotics, these compounds exhibit minimal environmental residues, lower toxicity, and reduced potential for resistance development [16]. Moreover, their multifunctional properties—ranging from antiviral and antibacterial activities to immune modulation—could make them significant in addressing the multifaceted challenges posed by pathogens like WSSV [17]. With this study, we aimed to investigate if a mixture of multiple organic acids (AuraAqua) can also prevent WSSV infection in vitro and alleviate the negative impact of infection in vivo. Moreover, we aimed to describe the biological mechanism by which the antimicrobial mixture exhibits its anti-viral effect and improves host immunity.

## 2. Materials and Methods

### 2.1. Virus, Antimicrobial Mixture and Shrimp Gut Primary Cell Isolation

White spot syndrome virus (WSSV) was propagated and isolated as previously described [18]. Post-larvae shrimps were purchased from a local hatchery in Bistrita, Romania and further cultivated in our research facilities as required in our experimental design. Ethical approval was not required for this study. After 48 h post-infection, the allantoic fluids were harvested using low-speed centrifugation and stored at −70 °C. Virus preparations were performed as previously described [19]. In our study, we used AuraAqua, a mixture of maltodextrin (4%) and sodium chloride (0.5%). As additives, lactic acid (30%), citric acid (15%), and citrus extract (8%) were also included and the balance to 100% was made up with dH_2_O. For primary cell preparations (SGPs), 5–10 *P. vannamei* gut tissue samples were harvested and prepared as previously described [20].

### 2.2. WSSV Quantification Using PCR

The virus quantification was performed as previously described [21]. Twenty milligrams of gut tissue from shrimp or infected SGP cells were homogenized in 500 μL of guanidine lysis buffer (50 mM Tris–HCl, 25 mM EDTA, 4 M guanidinium thiocyanate, 0.5% N-lauroylsarcosine, pH 8.0) at RT (room temperature). After centrifugation at 15,000× *g* for 3 min, 20 μL of silica was added to the supernatant for DNA absorption. Then, the mixture was spun for 5 min and centrifuged at 15,000× *g* for 30 s. The pellet was washed twice with 70% ethanol, resuspended in 20 μL dH_2_O, and centrifuged at 15,000× *g* for 2 min. WSSV-specific primers were used for PCR (forward primer 5′-tattgtctctcctgacgtac-3′ and reverse primer 5′-cacattcttcacgagtctac-3′). The parameters for PCR reactions were as follows: 5 min at 94 °C, 40 cycles at 94 °C for 45 s and 68 °C for 1 min, and extension at 68 °C for 5 min. For quantitative analysis of viral DNA, the real-time PCR was conducted. The TaqMan probe was 5′-FAM-tgctgccgtctccaa-TAMRA-3′. DNA standards were prepared as previously described [18] in order to generate a standard curve for absolute quantitation. Viral DNA was isolated and amplified using the above-mentioned primers at similar conditions. For generation of the standard curve, ten-fold serial dilutions of the DNA were made over a range of 8 log units (10^9^–10^2^) (efficiency: 98.64%; R^2^: 0.99). The results of three independent experiments are shown as mean ± standard deviation (SD). *p* values of <0.05 were considered significant.

### 2.3. Cytotoxicity Assay (CC_50_, EC_50_, and SI)

The AuraAqua mixture was used to determine the EC_50_ and SI (selective index). The mixture was titrated from 1 to 1:128 CC_50_ and used for virus contact. After the addition of 10 μL MTT reagent (Sigma Aldrich, Gillingham, UK), the samples were incubated for 4 h at 37 °C. The EC_50_ concentrations were calculated against Aq concentrations. To find out the cytotoxic activity of Aq, the antimicrobial product was dissolved in water and added to the SGP medium at concentration of 2% to quantify the host cell effect. The SGP cells were grown in 0.001–0.2% Aq containing medium. The experiment was repeated three times and on three different occasions. The absorbance of each well was recorded at 620 nm in a microplate reader (Fluostar Omega, BMG Labtech, Belfast, UK) to calculate the percentage of cell survival.

### 2.4. In Vitro Antiviral Activity

For antiviral activity, SGP cells were exposed to Aq before infection or infected with Aq pre-exposed virus. Following supernatant removal, the cells were washed and infected. In the case when the antimicrobial mixture was used to pre-treat the virus, the incubation took place for 1 h at room temperature followed by SGP cell infection. The absorption period was analyzed by mixing the viruses individually with 0.1%, 0.5%, and 2% Aq followed by infection of cells. For replication measurement, the inoculum was removed and replaced with media containing 0.5% methylcellulose. The experiment was repeated three times and at three different occasions. Data were subjected to statistical analyses using the Graph Prism software package version 11.

### 2.5. In Vivo Shrimp Challenge Test

Firstly, the in vivo activity of AuraAqua against WSSV was performed to test its activity in preventing *Penaeus vannamei* post-larvae survival. Twenty-five shrimp post-larvae per replicate were plated in sterile Petri dishes and intramuscularly inoculated into *P. vannamei* (shrimp infectious dose with 50% endpoint) per mL. The antimicrobial mixture was applied at the time of infection in concentrations of 0% and 2% in 5 L flasks. Survival was determined by counting the larvae at 2 days after infection. The first controls included an un-infected group and in the absence of Aq. The second control included WSSV-challenged shrimp in the absence of Aq. The experiment was performed in triplicate. Secondly, we infected shrimp with removed and non-removed peritrophic membrane. The removal of the peritrophic membrane was performed as previously suggested [19]. Shrimps were inoculated orally with 50 μL of a 10^−3^ WSSV stock. A total of 10 DNA samples was used for analysis from each experimental group. As previously described [22], the resulting infected and un-infected shrimp gut tissue was also used to quantify using qPCR the relative expression levels of mucin-1 (F ggctcggaagttggcgatgatg; R cgatggctcaatggcgaagagg), mucin-2 (F tgccagccacgtcctccttg; R ccgcagccgaggcagtcc), mucin-5AC (F agcaggacttcaacgactacaacag; R gcgcgacgccgatgatgg), mucin-5B (F cttgacgcatacgctcaggttcc; R tccgccgccttcatcctctg). and mucin-19 (F gaagaggaggaagaggacgaggag; R ggaccaccaggcacaagaacatc). The β-actin gene was used as reference gene (F gccctgttccagccctcatt; R acggatgtccacgtcgcact). The experiment was performed in triplicate. Quantification of LGBP gene expression using real-time RT-PCR was performed as previously described [23]. Briefly, the amplification was performed by using LGBP (β-1,3-glucan-binding protein) gene-specific primers (F ggtaaccagtacggaggaacga and R, tactcgacgtgggtcttctcga) from gut tissue cDNA, using an SYBR Green RT-PCR at 50 °C for 2 min and 95 °C for 10 min, followed by 40 cycles of 95 °C for 15 s and 60 °C for 1 min. As internal control, we used the actin gene (F-tcgccgaactgctgaccaaga and R, ccggcttccagttccttacc). The experiment was repeated three times. The levels of oxygenase expression (HO-1) were detected as previously described [24] in the shrimp gut. The primers used for HO-1 amplification were qHO-1-F gcatggcagtgaccgagattga and qHO-1-R gtcgctgcttcgtctcctcatc. The relative gene expression level for investigated genes was analyzed using the 2^−ΔΔCt^ method. The expression levels of LvECSIT were measured in gills at 2 days post-infection from each group (control, infected and infected + 2% Aq). The primers used were in this study were LvECSIT-F atgattcttatgaacgctt and LvECSIT-R aatttgggcatccagta [25]. β-actin was used as internal reference, and the PCR parameters were used as previously described [26]. The expression of carboxypeptidase B in the hepatopancreas and in the gut tissue of challenged shrimp and treated with 2% Aq was performed as previously described also using the primers CPB-real time-F gacatttcgtagaccatcacc and CPB-real time-R gaacttgccactatacagcgt [27].

### 2.6. Peritrophic Membrane (PM) Extraction and Isolation

*P. vannamei* ingesta free PMs were extracted as previously described [28]. Briefly, after 3 days of feed-free time, the PMs were suspended in 100 mL of 1% calcofluor (Sigma Aldrich, Gillingham, UK) containing protease inhibitors (Sigma, UK).

### 2.7. Measurements of H_2_O_2_, SOD, and CAT

The effect of AuraAqua on superoxide dismutase (SOD) and catalase (CAT) in WSSV-infected SGP cells or in challenged shrimp was measured as previously described in 10 samples [20]. SOD activity was established by means of a commercially available SOD colorimetric activity kit (Thermo Fisher, Horsham, UK) and CAT by using a catalase activity kit (Abcam, Trumpington, UK, ab83464). The methods were performed as indicated in the instruction manuals. Inhibitors of NADPH activity, including diphenyleneiodonium chloride (DPI, Sigma; 15 µM, 45 min preincubation and wash out) and bovine liver catalase (Sigma-Aldrich, Gillingham, UK; 300 U/mL), were used during the 24 h measuring interval. The SOD and CAT activity was measured in disrupted gut tissue using sonication for 60 s (4×) at 4 °C (in ice) in 1% saline solution and centrifuged at 2500 rpm at 4 °C for 5 min (Ultrawave DP200-00, Ultrawave Ltd., Cardiff, UK). Superoxide dismutase (SOD) and catalase (CAT) activity in WSSV-challenged shrimp was measured in the resulted supernatants. Hydrogen peroxide (H_2_O_2_) in the WSSV-infected SGP cells was measured using a PeroxiDetect™ Kit (Sigma-Aldrich, Gillingham, UK). All experiments were performed in triplicate.

### 2.8. IL 22 Expression Using qPCR and Western Blotting

To analyze the expression of IL-22 using qPCR, total RNA was extracted from infected SGP cells. The gut tissue was disrupted using sonication for 60 s (4×) at 4 °C (in ice) in 1% saline solution followed by centrifugation at 2500 rpm at 4 °C for 5 min (Ultrawave DP200-00, Ultrawave Ltd., Cardiff, UK). The RNA was extracted using the RNeasy Plus Mini Kit (Qiagen, Manchester, UK) and reverse transcribed using Transcriptor First Strand cDNA Synthesis Kit (Roche, East Sussex, UK). The mRNA levels were determined using quantitative RT-PCR using QuantiNovaSYBR Green PCR Kit (Qiagen, Manchester, UK) on a LightCycler 96 (Roche, East Sussex, UK). For quantification, primers IL-22-qF ccgtactgtagcaacagtgcag and IL-22-qR tcacattcttgcagagcaggattc were used [29]. The qPCR protocol included 1 × 95 °C for 30 s and 40 × 95 °C for 10 s, 60 °C for 20 s, and 72 °C for 20 s. Fold changes of expression were calculated by comparing the relative expression levels of the experimental groups with those of corresponding control groups, as previously described, also including detection using Western blotting [30]. For Western blotting, the anti-IL-22 antibody (P211) was purchased from Thermo Fisher Scientific, Manchester, UK. Briefly, following extraction, the proteins were separated in 15% SDS-polyacrylamide gel, transferred to nitrocellulose membrane using a 25 mM Tris buffer. The membranes were blocked with 5% milk in 20 mM Tris-HCl, 150 mM NaCl, 0.05% Tween 20, at pH 8.0 (TBST buffer) overnight at 4 °C. The next day, the membrane was incubated with IL-22 primary antibody overnight at 4 °C. The membranes were extensively in TBST buffer followed by horseradish peroxidase-conjugated secondary antibody (Thermo Fisher, Horsham, UK) for 2 h at room temperature. The membranes were washed three times with TBST and the blots were detected by using enhanced chemiluminescence reagent (ECL) and exposed to photographic films (Kodak, Thermo Fischer Scientific, Manchester, UK). The experiment was repeated three times.

### 2.9. Statistical Analysis

GraphPad Prism 10 software was used to perform statistical analyses. A *p* value < 0.05 was considered statistically significant following estimations using the Student’s *t*-test. An ANOVA was performed to analyze the percentage decrease between Aq concentrations compared to control using one-way ANOVA (*p* < 0.0001).

## 3. Results

### 3.1. The In Vitro Effect of Aq Against WSSV Replication and Ability to Infect SGP Cells

**Figure 1:**
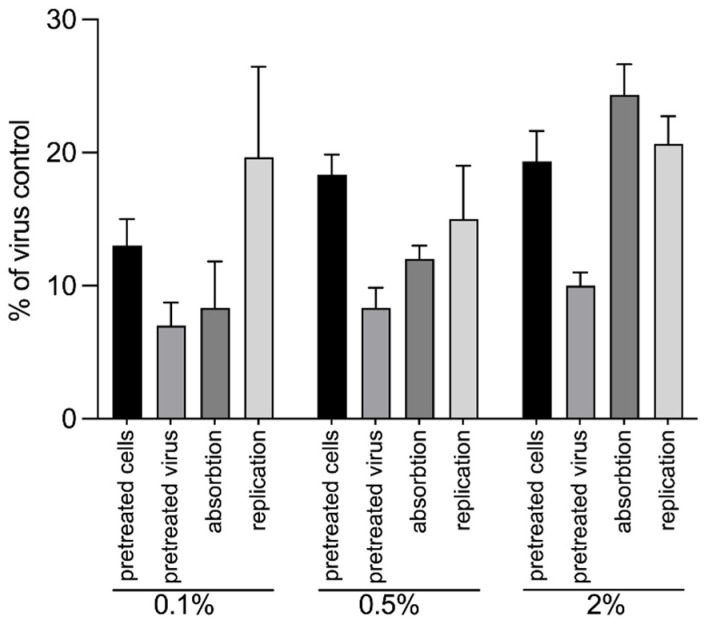
The anti-viral effect of AuraAqua against WSSV. AuraAqua (Aq) was added at the non-cytotoxic concentrations of 0.1%, 0.5%, and 2%. SGP cells were either pre-treated with Aq prior to WSSV infection, pre-treated with WSSV prior to infection, or Aq was included during the adsorption period (adsorption) or after penetration of the virus into cells (replication). One-way ANOVA (*p* < 0.0001) was used to analyze the data and significance of percentage decrease at the different concentrations.

The direct effect of Aq on WSSV replication was first investigated in vitro. The 50% effective concentration (EC_50_) of Aq was detected at 0.003 μL/mL (Table 1). Next, we aimed to demonstrate the effect of Aq on the WSSV replication by treating the SGP cells and the virus with Aq prior to infection, through their inclusion during adsorption or after adsorption. Infected SGP cells in the absence of Aq were used as a control (Figure 1). The pre-treatment of cells with Aq (0.1%, 0.5%, and 2%) led to a significant reduction in virus production compared to the control. Similar results were detected during viral replication, when Aq was included only in the cell media. Collectively, these results suggest that the anti-viral effect of AuraAqua is exerted through all phases of host cell infection. The concentration of 2% Aq was selected for further investigations.

**Figure 2:**
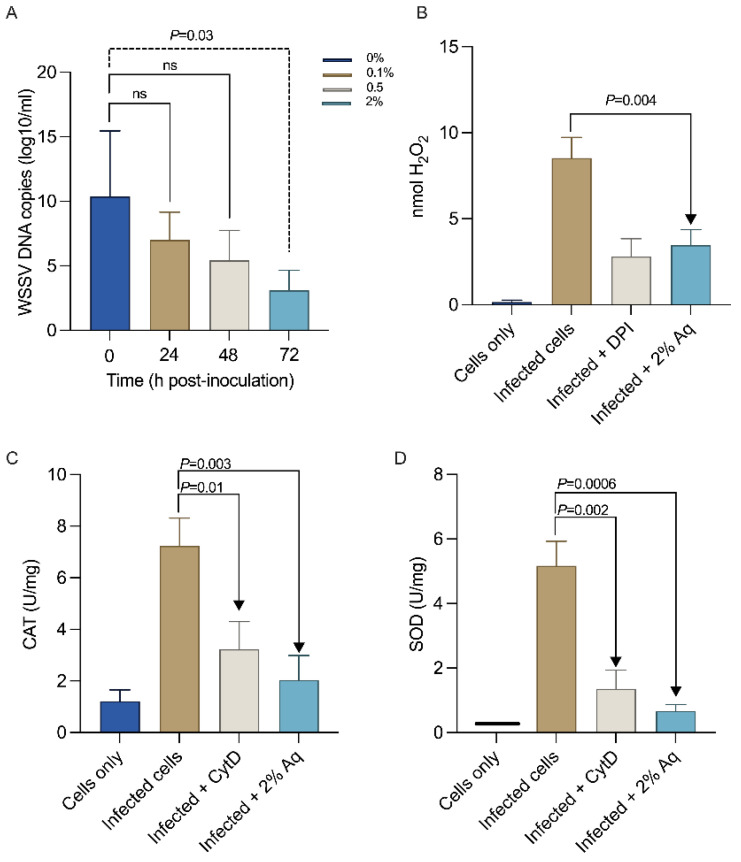
Time course of WSSV infection of SGP cells as assessed using qPCR (**A**) at 2% Aq. The WSSV DNA copies were measured at 27 °C at 0, 24, 48, and 72 h post-inoculation. H_2_O_2_ release (**B**), catalase (**C**), and superoxide dismutase (**D**). The results are presented as the mean of three independent experiments of the copy number of WSSV genomes in the WSSV-infected SGP cells incubated at 27 °C over time. The statistical significance was determined using Student *t*-test; *p* values are indicated on the graphs. (ns—not significant).

The percent reduction in WSSV replication was calculated relative to the amount of virus produced in controls, and a concentration of 2% was used further in all assays. The presence of 2% Aq during the in vitro infection assay gradually decreased the WSSV replication levels until they reached significance (*p* = 0.03) at 2% Aq after 72 h (Figure 2A). Additionally, we found that, at 72 h post-infection, there was a significant increase in oxidative stress as measured by the amount of H_2_O_2_ released by the infected SGP cells (Figure 2B). However, these levels were significantly reduced in the presence of 2% Aq (*p* = 0.004). Our results also showed that the addition of DPI during infection resulted in reduced levels of H_2_O_2_ being released due to their role in cellular NADPH inhibition. Both CAT (Figure 2C) and SOD (Figure 2D) activity were also decreased in the presence of 2% Aq, a consequence of decreased infection. Overall, these results show that Aq can prevent WSSV replication, cellular infection, and oxidation.

### 3.2. The Effect of 2% Aq in Preventing WSSV-Induced Mortality and Stimulating Mucin Production In Vivo and the Impact of PM

**Figure 3:**
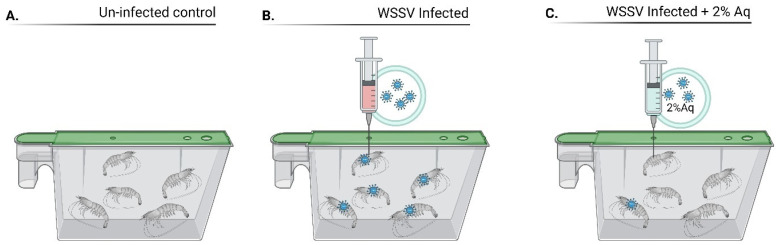
In vivo experimental design. The experiment included an un-infected and un-treated control (**A**); WSSV infected and un-treated (**B**); WSSV infected + 2% Aq (**C**). The experiment was performed for a period of 2 days and repeated three times. The experiment was also performed in the presence/absence of the peritrophic membrane (PM). Designed with Biorender.com.

To estimate the efficiency of Aq, we performed challenge studies by infecting *P. vannamei* shrimps with WSSV (Figure 3). The mortality rates decreased from 96.66%, recorded in the infected shrimp (wild-type gut) and in the absence of Aq, to 7.33% in the presence of 2% Aq (Table 2) (the average morality between the experiments is presented).

**Figure 4:**
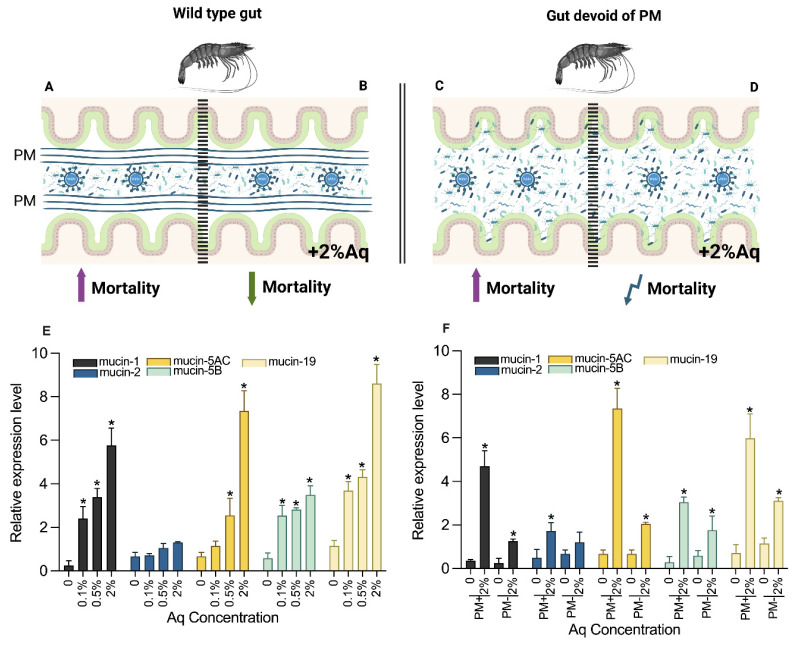
The impact of PM in mediating shrimp survival in the presence of Aq. Panels (**A**,**B**) (+2% Aq) indicate the experimental design in the infected wild-type shrimp. The design for the shrimp devoid of PM is presented in panels (**C**,**D**) (+2% Aq). Mucins gene expression levels in the intestines of wild-type *P. vannamei* at 2 days post-WSSV infection (**E**) and in the PM devoid gut (**F**). β-actin expression was used as reference. Vertical bars represent the mean ± SE (n = 3). Data indicated with asterisks (*) were significantly different (*p* < 0.05) among the two groups at the same time point. Panels (**A**,**B**) designed with Biorender.com.

The decrease in mortality was proportional with the increase in Aq concentration. Moreover, as indicated in Figure 4E, a significant increase (*p* < 0.05) in mucin-1, mucin-2, mucin-5AC, mucin-5B, and mucin-19 genes was also detected. These results clearly indicate that the antimicrobial mixture has the potential to protect the shrimp cultures in vivo.

Next, we aimed to explore if the peritrophic membrane (PM) has a role in mediating Aq efficacy. The data presented in Table 3 clearly indicate that in the absence of PM, the mortality rates decreased to only 27.8% compared to 9.34% in the presence of PM.

This lower survival rate in the PM group clearly indicates that PM has a role in mediating the efficacy of Aq. In addition, the results presented in Figure 4F show that in the absence of PM (at 2% Aq), the gut responds by significantly increasing the expression of mucin-1, mucin-2, mucin-5AC, mucin-5B, and mucin-19 genes, and potentially contributes to lower WSSV infection rates in shrimp (Figure 4F). These results show that under the influence of Aq, the shrimp gut structures improve their resilience by increasing the expression of mucins with a direct impact on shrimp survivability during WSSV infection.

### 3.3. The Impact of Aq on the WSSV Levels in the Shrimp Gut and on CAT and SOD Expression

**Figure 5:**
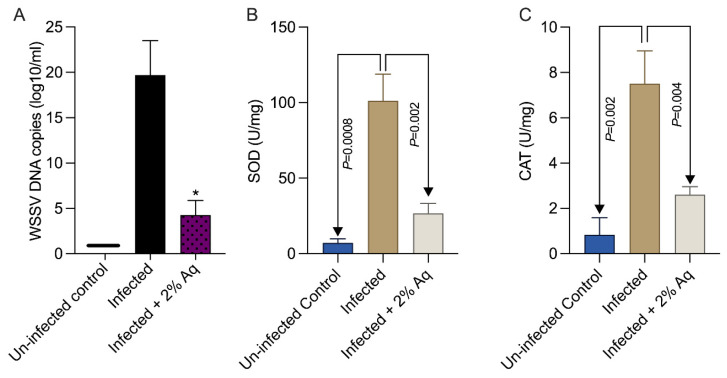
The WSSV (**A**), SOD (**B**), and CAT (**C**) levels in the shrimp gut tissue in the presence of 1% Aq. The results are presented as the mean of three independent experiments of the copy number of WSSV genomes in gut tissue at 2 days post-challenge. The statistical significance was determined using Student *t*-test; *p* values are indicated on the graphs. Data indicated with asterisks (*) were significantly different (*p* < 0.05) among the two groups at the same time point.

Our next goal was to measure the gastrointestinal levels of WSSV in the infected shrimp gut tissue. Clearly, as indicated in Figure 5A, the levels of WSS virus were significantly increased (*p* < 0.05) following infection of the un-treated group. However, in the presence of 2% Aq, the infection levels dropped significantly (*p* < 0.05). This reduction in virus load in the presence of 2% Aq was also associated with a significant reduction in SOD (Figure 5B) and CAT (Figure 5C) production in the infected shrimp. Firstly, these results confirmed the initial in vitro results indicating a decreased infection rate in the presence of 2% Aq. Secondly, the antioxidant role of CAT or SOD has become unnecessary in the presence of Aq, probably due to the decreased infection rate mediated via a separate mechanism.

### 3.4. The Impact of Aq on the Expression of LGBP and HO-1 Proteins

**Figure 6:**
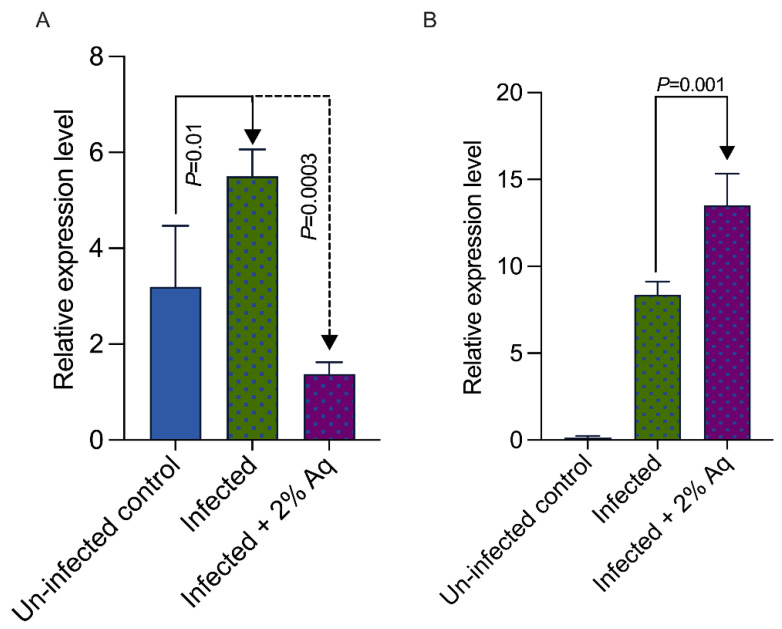
β-1,3-glucan-binding protein (LGBP) (**A**) and oxygenase (HO-1) (**B**) gene expression levels in the intestines of *P. vannamei* at 2 days post-WSSV infection. β-actin expression was used as reference. Vertical bars represent the mean ± SE (n = 3).

To further explore the impact of Aq in mitigating WSSV-induced oxidative stress, we measured the expression of β-1,3-glucan-binding protein (LGBP) and the antioxidant protein HO-1 oxygenase in the gut tissue. The expression of the WSSV interaction protein, LGBP (Figure 6A), was significantly reduced (*p* = 0.0003), while the expression of the antioxidant protein, HO-1 (Figure 6B), exhibited a significant increase (*p* = 0.001). These results confirm that the reduction in WSSV infection was caused by Aq, firstly by blocking the cellular entry mechanisms and secondly via an increased antioxidant mechanism to reduce the impact of infection-generated oxidative stress.

### 3.5. The Effect of Aq on the LvECSIT Expression in WSSV-Challenged Shrimp

**Figure 7:**
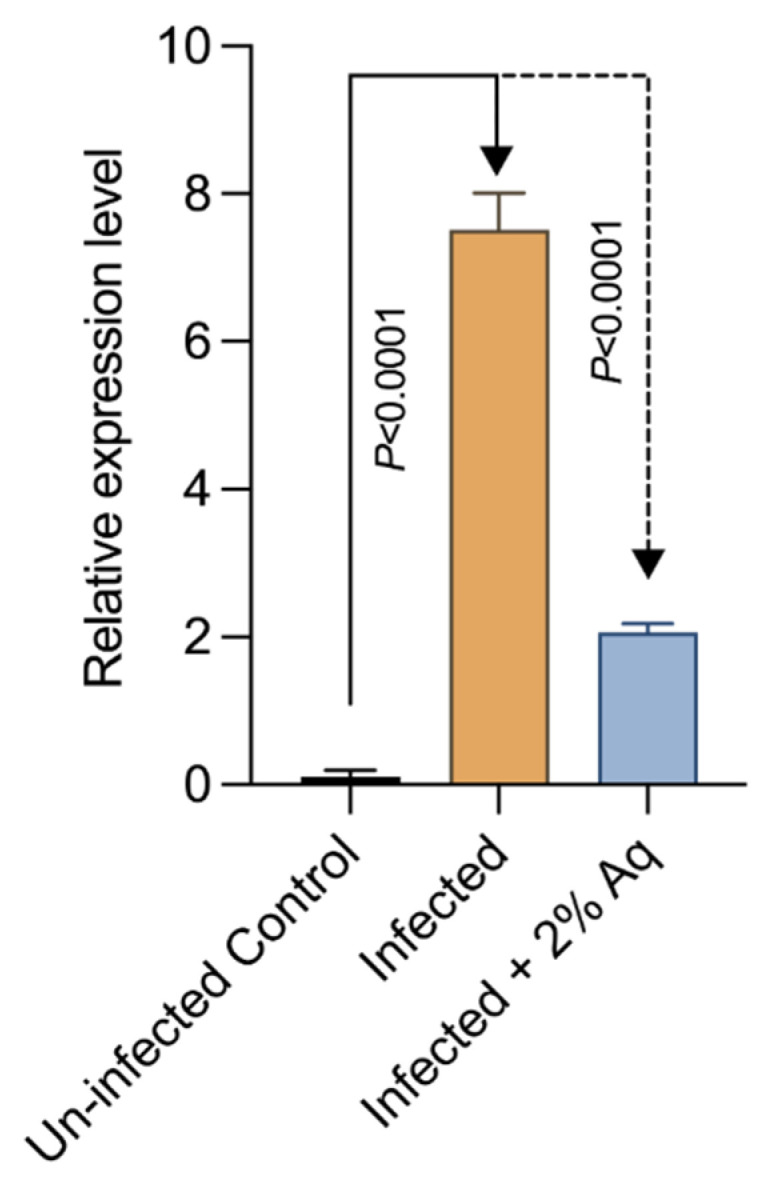
LvECSIT expression in the shrimp gills in the presence of 0.2% Aq at 2 days post-WSSV infection. β-actin expression was used as reference. Vertical bars represent the mean ± SE (n = 3). The statistical significance was determined using Student *t*-test; *p* values are indicated on the graphs.

Moreover, we aimed to further characterize the involvement of Aq in mediating the interaction between WSSV and the shrimp immune system. To gain an insight, we measured the expression levels of the evolutionarily conserved signaling intermediate in toll pathways (LvECSIT) in gills. As indicated in Figure 7, the expression of LvECSIT was significantly upregulated in infected shrimp (*p* < 0.0001), and this trend was reversed in the presence of Aq (*p* < 0.0001). These results clarify and confirm that Aq reduces the need for the host’s innate immunity activation due to the reduction in WSSV infection rates.

### 3.6. The Impact of Aq on IL-22 Cytokine Production in WSSV-Infected Shrimp

**Figure 8:**
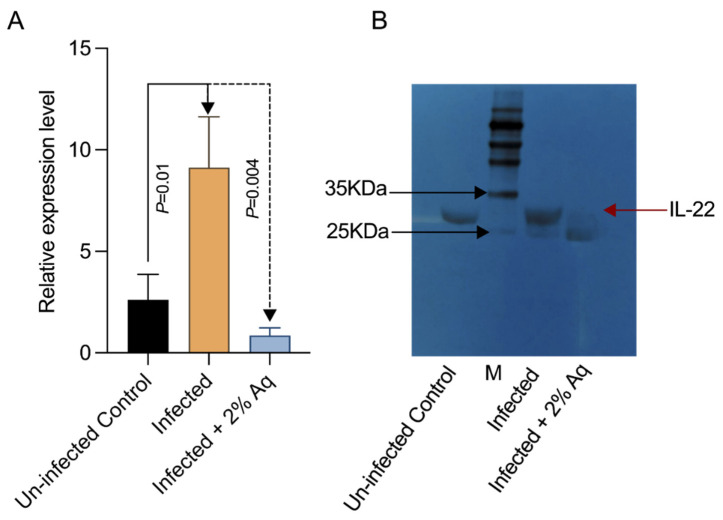
The shrimp Interleukin 22 (IL-22) relative expression levels as detected using qPCR at 2 days post-infection (**A**,**B**) Western blot analysis of the IL-22 protein expression levels of WSSV-challenged shrimp. The un-infected shrimp and un-treated shrimp were used as controls. Shrimp β-actin was used as loading control. M, protein marker. Statistically significant differences are indicated on the graph (**A**).

To further prove the impact of Aq in mitigating the consequences of WSSV infection, we next measured the levels of IL-22 production, otherwise known as one of the responders to WSSV infection in shrimp. Our results (Figure 8A) confirmed the increase in IL-22 production in infected shrimp, followed by a significantly lower detection in the presence of 2% Aq. The decrease in gene expression was also confirmed when the amount of protein produced was also estimated using Western blotting (Figure 8B), indicating increased IL-22 levels in the infected shrimp and lower protein levels in the infected shrimp in the presence of 2% Aq.

### 3.7. The Impact of 2% Aq on the Expression of Carboxypeptidase B in the Shrimp Gut

**Figure 9:**
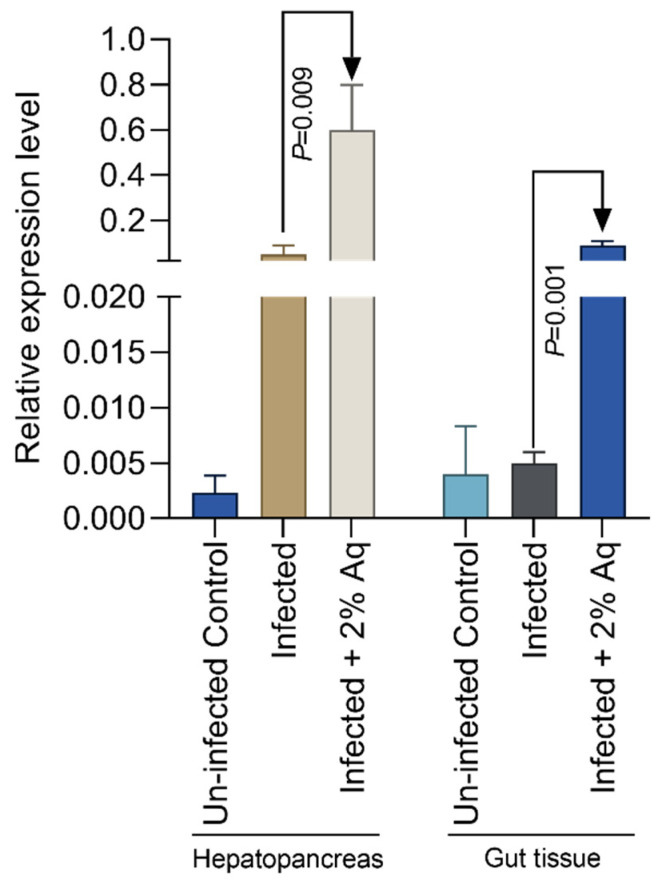
Carboxipeptidase B expression in the hepatopancreas and the gut tissue in challenged shrimp. The relative expression levels as detected using qPCR at 2 days post-infection in WSSV-challenged shrimp. The un-infected shrimp and un-treated shrimp were used as controls. Shrimp β-actin was used as loading control. M, protein marker. Statistically significant differences are indicated on the graph.

We used the expression of carboxypeptidase B to further prove the impact of Aq in reducing WSSV infections in shrimp. This proteolytic enzyme, which is usually downregulated in WSSV infected shrimp, exhibited a significantly recovered expression in both the hepatopancreas (*p* = 0.009) and in the gut tissue (*p* = 0.001) of WSSV-challenged shrimp (Figure 9). This impact on the carboxypeptidase B expression also suggests that Aq is involved in maintaining gut health and improve feed digestibility in shrimp due to its role in the degradation of proteins involved in the formation of peptides and hormones.

## 4. Discussion

White spot syndrome virus (WSSV) is responsible for significant economic losses in the shrimp industry by triggering the white spot disease (WSD) [31]. Finding and designing efficient on-farm interventions should focus on the virus only, since the host gut seems to be unaffected in WSSV-infected shrimp [32]. Mixtures of organic acids have been shown before to be effective in preventing viral infections both in vitro and in vivo in farmed animal models [17]. This type of intervention, other than vaccination, could be more effective in the long-term, given that recent data on WSSV outbreaks indicate that genetic variations can contribute to WSSV virulence and disease progression in shrimp [33].

Multiple host-entry pathways are employed by WSSV, for which the most common route is oral ingestion. VP28 is an integral viral envelope protein mediating host cell receptor interactions, entry, and systemic dissemination [9]. After infection, the process of viral hijacking of cellular machinery propagates, rendering metabolic reprogramming in immune cells as well as other target tissues [34]. Among these, glycolytic enzymes and the pentose phosphate pathway are necessary for providing the nucleotide precursors required for viral replication [34]. Additionally, these pathways supply nucleotide and lipid substrates that viruses utilize for biosynthesis [7]. Furthermore, lipid metabolism is upregulated to produce the components necessary for forming viral membranes, all providing substrates essential for viral replication and morphogenesis [7,35].

The peritrophic membrane (PM) provides protection to the gastrointestinal tissue and cells while simultaneously allowing the penetration of nutrients, water, and minerals into the host [36]. These characteristics give the PM an important role for both gut immunity and digestion in invertebrates [37]. Structurally, the PM in shrimp is known to include the lipopolysaccharide β-1,3-glucan-binding protein (LGBP) [28], which can act as a pathogen recognition protein and is involved in the activation of the shrimp immune system. Critically for our study, this protein plays an important role in the interaction of WSSV with the host in shrimps [23]. The current results showed that when shrimp were devoid of PM and infected with WSSV, this resulted in higher mortality rates (27.8%) when compared to PM intact shrimp (9.33%) in the presence of 2% Aq. However, these reductions in mortality rates in both PM− and PM+ shrimp were only possible in the presence of Aq, since in the un-treated shrimp groups the mortality rates were over 97%. This reduction in mortality can also be explained by the increase in mucus production and a reduced expression of the LGBP gene, which might explain the attenuated infection in the presence of Aq. The inhibitory effects of natural products (plant extracts) against β-1,3-glucans and their biosynthesis pathways were previously described, including their chitin inhibitory effects, another component of PM [38]. It is very possible that the observed decrease in LGBP expression was a secondary effect caused by the reduced virus load levels. Moreover, Aq was responsible for the decline in host oxidative stress (both in vitro and in vivo), as indicated by the reduced H_2_O_2_, CAT, and SOD levels in the presence of 2% Aq. Oxygenase, also identified in the PM (Heme oxygenase-1 HO-1), is usually induced to mitigate the damage caused by the WSSV-induced oxidative stress [24]. Our data clearly indicated that in WSSV-challenged shrimp, the gut HO-1 expression levels were significantly increased in the presence of 2% Aq.

The immune response in WSSV-infected shrimp is triggered via the LvECSIT, an evolutionarily conserved signaling intermediate in Toll pathways, which plays an important role in the innate immunity [39] and defense against WSSV [25]. In the presence of Aq, and at low infection levels, the expression of LvECSIT was detected at significantly low levels in gills. Interleukin-22 (IL-22), one of the cytokines upregulated in WSSV-infected shrimp [30], was detected in our study at very low levels both in gene and protein expression in the presence of Aq. Additionally, carboxypeptidase B, a proteolytic membrane involved in breaking peptide bonds at the amino-acid C terminus, has been marked as downregulated as a response to WSSV infection [40]. At low infection levels, in the presence of 2% Aq, this enzyme was significantly upregulated in the gut tissue (*p* = 0.001) and hepatopancreas (*p* = 0.009) of the challenged shrimp. These results suggest that Aq is involved in maintaining gut health in shrimp exposed to WSSV infection. The role of carboxypeptidase B in nutrition was also highlighted in other species, such as poultry, where this enzyme was found to be critical in the ileal digestion of proteins [41].

In addition to metabolic disruption, WSSV compromises the shrimp immune system by altering the expression of immune-related genes and metabolites. For instance, linoleic acid levels significantly increase post-infection, serving dual roles in antiviral immunity and viral replication inhibition [42]. WSSV also modulates lipid metabolism in haemocytes and the hepatopancreas, alternating between lipolysis and lipogenesis to meet its energy necessity and support virion assembly [35,42]. The innate immune system of shrimp plays a pivotal role in their defense versus WSSV as well as other pathogens. WSSV undermines the shrimp’s immune system by reprogramming metabolic pathways and suppressing immune-related genes. The virus’s rapid replication cycle (~24 h) and ability to modulate host immune responses make it exceptionally challenging to control [34]. Despite the absence of adaptive immunity in shrimp, innate immune mechanisms, such as AMPs and reactive oxygen species (ROS) production, melanization, phagocytosis, and apoptosis, provide limited defense [7,34]. Key enzymes, such as superoxide dismutase (SOD), catalase (CAT), and alkaline phosphatase (AKP), are integral to the shrimp’s defense system [8]. SOD, for instance, mitigates oxidative stress by converting harmful superoxide radicals including ROS into hydrogen peroxide, which is subsequently broken down into water and oxygen by CAT [8]. Studies showed that shrimps fed with low concentrations of cup plant (*Silphium perfoliatum* L.) have demonstrated the ability to counter the deleterious effects of WSSV and *Vibrio parahaemolyticus*, or even improve the resistance of shrimp by providing strong growth of beneficial intestinal bacteria and enhancing significantly the immune-related enzymes (SOD, CAT, AKP, LZM, α-AL and LIP) and modulated immune-related genes (*Crustin*, *Lysozyme, LGBP*, *LvLec*, *proPO* and *PEN-3α*) from the hepatopancreas and intestine [43]. According to such observations, this not only improves the host’s immune capacity, but also maintains the integrity of important tissues, reducing the susceptibility to WSSV.

## 5. Conclusions

**Figure 10:**
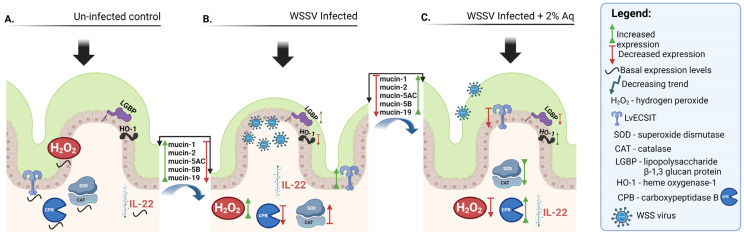
The molecular mechanisms of Aq and its role in preventing WSSV infection in vivo in a shrimp challenge model. The role of Aq in the un-infected and un-treated control (**A**), in WSSV infected shrimp (**B**), and in WSSV-infected shrimp in the presence of 2% Aq (**C**). Design created with Biorender.com.

This study demonstrates the potential of a mixture of organic acids to attenuate WSSV infections in shrimp using in vitro and in vivo infection models. Our results showed that Aq exerts its anti-WSSV activity through multiple mechanisms, including the direct inhibition of viral replication across all phases of host cell infection, the modulation of oxidative stress responses, and the enhancement of innate immune pathways. In vivo experiments illustrated in Figure 10 further corroborate the protective effects of Aq in WSSV-challenged shrimp, reducing mortality rates significantly from 96.66% in un-treated shrimp to just 7.33% with 2% Aq.

The treatment-mediated reductions in H_2_O_2_ emphasize its antioxidant properties, which mitigate WSSV-induced cellular damage. Additionally, regulating immune responses by including the downregulation of IL-22 and LvECSIT in infected shrimp accentuates its role in tempering inflammatory and immune activation, while effectively controling viral replication. The upregulation of CPB in gut and hepatopancreas tissue further underlines a resulting contribution to gut health and protein digestion, evidencing shrimp resilience against infection. By addressing WSSV infections via antiviral, immunomodulatory, and antioxidative mechanisms, such alternatives could offer a mitigating resolution to managing pathogens. Future research could further investigate the effect of water quality on the treatment efficiency and the long-term health effects. The resulting findings could pave the way to incorporate natural antimicrobials into sustainable aquaculture practices, the potential implications of which extend to other viral and bacterial diseases in farmed species.

## Figures and Tables

**Table 1:** Cytotoxicity and efficiency of AuraAqua.

Virus	CC_50_ (µL/mL)	EC_50_ (µL/mL)	SI
WSSV	0.21	0.003	70

CC, cytotoxic concentration; EC, effective concentration; SI, selective index (CC_50_/EC_50_).

**Table 2:** Mortality of *Penaeus vannamei* 2 days post-challenge after infection with WSSV.

AuraAqua Concentration (%)	% of Mortality
0	96.66
0.1	54
0.5	28
2	7.33

**Table 3:** Mortality of *Penaeus vannamei* 2 days post-challenge after infection with WSSV and in the presence/absence of PM.

AuraAqua Concentration (%)	% of Mortality (PM+)	% of Mortality (PM−)
0	100	97.4
0.1	63	85
0.5	18	67.3
2	9.34	27.8

## Data Availability

Data available on reasonable request and not being subjected to ethical restrictions.
